# Phenolic Analysis and In Vitro Biological Activity of Red Wine, Pomace and Grape Seeds Oil Derived from *Vitis vinifera* L. cv. Montepulciano d’Abruzzo

**DOI:** 10.3390/antiox10111704

**Published:** 2021-10-27

**Authors:** Adriano Mollica, Giuseppe Scioli, Alice Della Valle, Angelo Cichelli, Ettore Novellino, Marta Bauer, Wojciech Kamysz, Eulogio J. Llorent-Martínez, Maria Luisa Fernández-de Córdova, R. Castillo-López, Gunes Ak, Gokhan Zengin, Stefano Pieretti, Azzurra Stefanucci

**Affiliations:** 1 Department of Pharmacy, G. d’Annunzio University of Chieti-Pescara, 66100 Chieti, Italy; a.mollica@unich.it (A.M.); giuseppe.scioli@unich.it (G.S.); alice.dellavalle@unich.it (A.D.V.); 2 Department of Medical, Oral and Biotechnological Sciences, “G. d’Annunzio” University Chieti-Pescara, Via dei Vestini 31, 66100 Chieti, Italy; angelo.cichelli@unich.it; 3 NGN Healthcare, Via Nazionale Torrette, 207, 83013 Mercogliano, Italy; ettorenovellino09@gmail.com; 4 Department of Inorganic Chemistry, Faculty of Pharmacy, Medical University of Gdànsk, 80-416 Gdànsk, Poland; marta.bauer@gumed.edu.pl (M.B.); wojciech.kamysz@gumed.edu.pl (W.K.); 5 Department of Physical and Analytical Chemistry, Faculty of Experimental Sciences, University of Jaén, E-23071 Jaén, Spain; ellorent@ujaen.es (E.J.L.-M.); mferna@ujaen.es (M.L.F.-d.C.); rcl00041@red.ujaen.es (R.C.-L.); 6 Department of Biology, Science Faculty, Selcuk University, Konya 42130, Turkey; akguneselcuk@gmail.com (G.A.); gokhanzengin@selcuk.edu.tr (G.Z.); 7 National Center for Drug Research and Evaluation, Italian National Institute of Health, Viale Regina Elena 299, 00161 Rome, Italy; stefano.pieretti@iss.it

**Keywords:** antioxidants, enzyme inhibition, grape pomace, seeds, phenolic Compounds, anti-inflammatory potential, antimicrobial activity

## Abstract

Grape pomace is commonly considered a waste product of monovarietal red wine production. Methods: HPLC-DAD analysis was performed to determine the polyphenol and flavonoid contents of all the extracts obtained from Montepulciano d’Abruzzo red wine and grape skins whereas, GC-MS was applied to the determination of fatty acid composition in grape seeds oil. Biological characterization involves antioxidant and antimicrobial assays for all the extracts and seeds oil; Their ability to inhibit α-glucosidase, α-amylase, α-tyrosinase, and ChE enzymes was also detected, together with anti-inflammatory activity on wine, grape skin extracts, and seeds oil by lipoxygenase (5-LOX) and LPS-stimulated macrophage release assays. Data indicate significative polyphenols content (199.31 ± 7.21 mgGAE/g), antioxidant (CUPRAC assay (1036.98 mgTE/g)), enzymatic inhibition (α-tyrosinase: 151.30 ± 1.20 mgKAE/g) and anti-inflammatory activities for wine-organic extract 2, while the antimicrobial activity of grape skin decoction is higher than those reported by wine extracts on three bacterial strains. Interestingly only dealcoholized wine and wine-aqueous extract exerts inhibitory effects on α-glucosidase (20.62 ± 0.23 mmolACAE/g and 19.81 ± 0.03 mmolACAE/g, respectively), while seeds oil is rich in oleic and linoleic acids. These results confirm the strong antioxidant properties of Montepulciano d’Abruzzo grape pomace, suggesting the potential use of this waste product as functional food supplements in the human diet and in cosmeceutics.

## 1. Introduction

*Vitis vinifera* L. is a typical crop cultivated in the Mediterranean area and is appreciated for its peculiar flavor and sensorial properties; it is a rich source of bioactive Compounds involved in the defensive mechanisms against Alzheimer, diabetes mellitus, and cardiovascular diseases [1,2]. The most common polyphenolic Compounds are flavonoids and phenolic acids, divided into hydroxybenzoic and hydroxycinnamic acids. Phenolic Compounds, as volatile precursors, contribute to sensorial properties such as color, flavor, and wine’s olfactory profile. Anthocyanins are responsible for wine and grapes color; malvidin, cyanidin, peonidin, delphinidin, petunidin, and pelargonidin are abundant, and the concentration of each depends on the varietal, viticultural practices, and environmental factors such as climate conditions and cultivation area [2]. The flavan-3-ols catechin, epicatechin, epigallocatechin, and procyanidins B1, B2, and B3 in wines and grape skins are associated with taste, antioxidant and antimicrobic activities [3,4,5,6]. Grape pomace is the solid residue of pressed grapes. It contains the skins, pulp, seeds, and stems of the fruit. Today this waste is mostly used as fodder, as fertilizer, or to make pallet fuels. Around 25% of grape weight from *Vitis vinifera* L. is composed of skins and seeds, considered as abundant by-products rich in anthocyanins and natural coloring agents [7]. The use of grape pomace for alternative applications has been inefficient for a long time due to a large portion discarded in landfills; furthermore, its market potential as a supplement decreases with its inclusion in compost and re-cyclization into the vineyard. Many papers report the investigation of biological properties and phytochemical profiles of red wine and grape skin, while grape seed extracts are little explored [8]. A recent study shows that grape seed increases the production of vascular endothelial growth factors in the wound healing process; furthermore, the inclusion of grape seed extract in a diet rich in calcium promotes bone formation and reinforcement in osteoporosis conditions [9,10]. Proanthocyanidins in grape seeds may prevent skin cancer development due to the reduction in oxidative stress and alteration of cytokine activity [11]. This cell-protective effect is highly desirable in the treatment of Alzheimer’s disease [12]. The National Center for Complementary and Integrative Health (NCCIH) documented a decrease in systolic blood pressure and heart rate, probably through the effect of antioxidants in grape seed extracts that can preserve blood vessels from damages [13]. Furthermore, grape seed extracts might be crucial in the management of various human conditions, such as: tooth decay, protection against pathogens, with improvement of night vision, diabetic retinopathy, blood sugar control, alleviate chronic venous insufficiency, anti-aging properties, and reduction in edema and iron levels in hemochromatosis [12,14]. Moreover, in times of increasing resistance to antibiotics, searching for natural Compounds with antimicrobial activity is needed. There are many potential ways of using phenols from wine both in the food industry and medicine. Wine extracts are tested as substances that can replace the sulfites in wine production to protect against bacteria. They can also be used as natural and antimicrobial ingredients of oral hygiene products, e.g., in liquid mouthwashes [15,16]. This plethora of beneficial properties and the potential application of diverse by-products from grapes prompted us to investigate the biological activities of different extracts from the pomace of Montepulciano d’Abruzzo red wine. Montepulciano wine is mostly produced in the central part of Italy, in the Abruzzo region; the most appreciated are DOC Montepulciano d’Abruzzo and the DOCG Colline Teramane wines in the Marche region. Montepulciano grapes have been largely used as bulk grapes in order to confer color and body structure to different monovarietal wines. The richness and complexity in aroma and taste are due to the identities and quantity of their volatiles, polyphenols, and alcohols [17]. Nowadays, only a few studies are reported in the literature on this typical product of the Abruzzo region [18,19]; thus, in this work, we focused our attention on Montepulciano red wine in order to better understand its healthy properties, antimicrobial and antioxidant activities. Recently the ability of polyphenols to inhibit the growth of trimethylamine N-oxide (TMAO)-producing bacterial strains has been described for grape pomace polyphenol extract, which is an important aspect, considering that TMAO is a risk factor for cardiovascular diseases [20]. The polyphenol and flavonoid composition in different extracts of red wine, grapes skin, and seeds were compared and analyzed in order to highlight the importance of the extraction process in the determination of the quantity and quality of these Compounds. The aim of this work is to validate the nutraceutical potential of Montepulciano d’Abruzzo red wine and grape skin extracts by determining their antimicrobial, anti-inflammatory, antioxidant, and enzyme inhibition activities.

## 2. Materials and Methods

### 2.1. Wine, Grape Skin, and Seeds Samples

**Figure 1:**
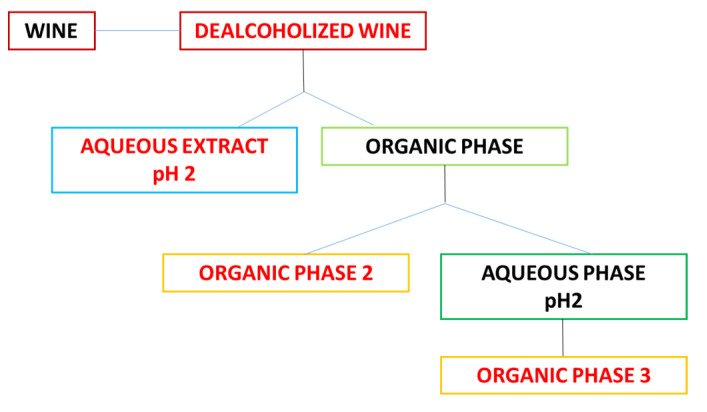
Extraction phases for red wine fractions.

The Montepulciano grape pomace was collected in 2019, the red wine was bottled from the same grape and analyzed one year later at Bolognano town territory (LAT. 42°13′49.0″ N/LON. 13°57′22.6″ E), in the province of Pescara in Abruzzo region. The grape pomace was lyophilized, the skins, pulp, and stems were separated from the seeds manually and then extracted with three different methods, e.g., decoction, microwave-assisted decoction, and soxhlet extractions, while seeds were used for the preparation of decoction in *n*-hexane. The red wine was extracted following the procedure previously reported by Ghiselli et al. [21] (Figure 1).

The freeze-dried grape pomace was separated into skin/pulp and seeds and powdered with a blender. The wine was stored in the dark at 15 °C until analysis. Then, it was opened and purged with nitrogen. Finally, it was closed with the original cork.

### 2.2. Preparation of Extracts for Analyses

The red wine extracts were prepared following the procedure previously reported by Ghiselli et al. [21].

First, the alcohol was removed under vacuum at 25 °C and 30 mbar, obtaining the first fraction, namely the dealcoholized wine. The dealcoholized wine (100 mL) was adjusted at pH = 2 with HCl 2N and extracted with ethyl acetate (EtOAc) three times (100 mL each), using centrifugation to avoid the formation of emulsion. The aqueous residue called wine-aqueous extract has been lyophilized, and the organic phase dried using vacuum. The dried organic phase was dissolved in deionized water at pH 7.0 and extracted with EtOAc (3 × 100 mL each), obtaining organic phase 2, which was dried under vacuum. The aqueous phase was adjusted at pH 2 with HCl 2N and extracted with EtOAc (3 × 100 mL each) to yield the organic phase 3 (Figure 1). Extracts of *Vitis vinifera* L. grape skins were prepared following three different techniques: Soxhlet, microwave, decoction (60% MeOH/H_2_O) extractions. The seeds were crushed with a clamp and extracted with *n*-hexane. All four extracts were lyophilized and tested for enzymatic inhibition and antioxidant properties. HPLC analysis was performed in red wine samples following the chromatographic conditions previously reported in the literature [22,23].

For the preparation of grape pomace extracts and seeds oil, 500 mg of freeze-dried material or crushed seeds were used following the procedure reported by Della Valle et al. [24].

### 2.3. Determination of Total Phenols and Flavonoids Content

The total phenolic and flavonoids content of dealcoholized wine, red wine/grape skin extracts, and seeds oil was determined using the AlCl_3_ method for flavonoids, and a solution of diluted Folin–Ciocalteu reagent for phenols following the procedures reported in the Supplementary Materials and previously applied [25,26]. In the determination of total phenolic, a calibration curve was drawn for gallic acid, and the results were expressed as gallic acid equivalent (a calibration curve from different concentrations (0–5 µg), Absorbance: 0.2199 (µg gallic acid), R^2^ = 0.9993). In the determination of total flavonoid content, a calibration curve was drawn for rutin, and the results were expressed as rutin equivalent (a calibration curve from different concentrations (0–20 µg), absorbance: 0.1274 (µg rutin) + 0.0506, R2 = 0.9968).

### 2.4. Chromatographic Analyses of Extracts

Chromatographic analyses conditions and instrumentation are reported and described in the Supplementary Materials.

#### Identification of Individual Phenolic Compounds by HPLC-ESI-MS Method

The HPLC technique was applied in combination with the ESI-MS method to identify the most interesting phenolic Compounds, as reported in the Supplementary Materials.

### 2.5. HPLC-DAD Resveratrol Quantification

Dealcoholized wine, grape skin microwave, grape skin decoction, and grape skin soxhlet extracts were analyzed for the quantitative determination of *trans*-resveratrol by HPLC-DAD analysis as reported by Cvetkovic et al., with some modifications [27]. HPLC analysis was performed using Waters XBridge TM Prep BEH C18-bonded 4.6 × 150 mm, at a flow rate of 1 mL/min, with an isocratic gradient of MeOH (0.1% TFA) for 15 min. The stock solution of *trans*-resveratrol was prepared by dissolving 1.2 mg of the standard Compound in 2.5 mL of MeOH. The sample was stored in the dark to avoid oxidative degradation and isomerization of *trans*-resveratrol. A total of 1 mL of stock solution was diluted to 10 mL with mobile phase (dil. 1), then 20 µL of the solution was injected and analyzed in HPLC at 340 nm. The concentration range was 0.005–5 μg mL (r 0.99). A total of 1 mL of dil. 1 was diluted to 10 mL with the mobile phase (dil. 2), then 20 µL of the solution was injected and analyzed in HPLC at 340 nm. Finally, a third dilution was prepared using 1 mL of dil. 2, transferred into a 10 mL graduated cylinder and filled to the mark with mobile phase (dil. 3); 20 µL was analyzed in HPLC at 340 nm (*n* = 3). *Trans*-resveratrol identification has been obtained using C 18 column (150 × 4.6 mm) and methanol as mobile phase. Maximum absorption of *trans*-resveratrol was detected at 324 nm with a retention time of 2.53 min.

The peak areas and the µg concentration of samples were graphed to provide the calibration curve (Figure S2, see Supplementary Materials) with the following equation:

A_324_ = 6 · 10^6^ + 22,873

The samples stock solutions were prepared using 2.5 mg of dealcoholized wine, grape skin microwave, grape skin decoction, and grape skin soxhlet extracts dissolved in 2.5 mL of methanol, then a volume of 20 µL was injected for each sample.

### 2.6. Determination of Fatty Acid Methyl Ester (FAME) Profile in Grape Seed Oil

The seed oil extract was analyzed by GC-FID technique following a standard procedure [28]. Fatty acid identification was performed by comparing commercial FAME standards (AccuStandard, New Haven, CT, USA) relative retention times. The results were given as FID response area in relative percentages, as mean and standard deviation of three GC replications.

### 2.7. Biological Assays

#### 2.7.1. Antioxidant Assays

In this work, the antioxidant properties of Montepulciano extracts were evaluated using six different methods (see Supplementary Materials). Antioxidant activities of dealcoholized wine, red wine/grape skin extracts, and seeds oil were determined using the method previously described by Savran et al. [29]. Trolox (for radical scavenging, reducing power, and phosphomolybdenum assays) and EDTA (for metal chelating) were used as positive controls, and the results were expressed as standard equivalents.

#### 2.7.2. Enzyme Inhibitory Assays

The ChE, α-amylase, α-glucosidase, and tyrosinase inhibitory activity was measured following the procedures reported in the supporting information (Supplementary Materials) and previously described in the literature [30]. Standard inhibitors were used as positive controls (galanthamine for cholinesterases, acarbose for α-amylase and α-glucosidase, and kojic acid for tyrosinase). The results of the enzyme inhibitory effects were expressed as equivalents of the standards.

#### 2.7.3. Antimicrobial and Antifungal Activity

Antimicrobial properties were tested according to the protocol described by CLSI (2012) [31], while antifungal activity was examined according to the CLSI protocol (2002) [32]. Assays were performed using reference strains of bacteria: *Staphyloccocus aureus* ATCC 25922, *Staphylococcus epidermidis* ATCC 2532, *Escherichia coli* ATCC 25922, and fungus: *Candida albicans* ATCC 10331 obtained from Polish Collection of Microorganisms (Polish Academy of Sciences, Wroclaw, Poland). A broth microdilution method was conducted using 96-wells plates and Mueller Hinton II broth for bacteria, and RPMI-1640 for fungus. Increasing concentrations of tested extracts (1–512 µg/mL) were used. The MIC is the concentration of Compound requested to induce the visible microorganism’s growth inhibition.

#### 2.7.4. In Vitro Cell Assay

Human peripheral blood monocytic cell line THP-1 was purchased from the American Type Culture Collection (Bethesda, Rockville, MD, USA), with the aim to evaluate the anti-inflammatory activity on LPS-stimulated macrophages. For further information, see supporting information (Supplementary Materials).

#### 2.7.5. Lipooxygenase (5-LOX) Inhibition Assay

The LOX inhibitor screening assay kit was used to evaluate each extract at 10 and 100 μg/mL concentration in triplicates, following the manufacturer’s protocol (Cayman test kit-766700, Cayman Chemical Company, 1180 East Ellsworth Road, Ann Arbor, MI 48108, USA). Experimental details are in the Supplementary Materials.

### 2.8. Statistical Analysis

Statistical analysis procedure for biological experiments is described in the Supplementary Materials.

## 3. Results and Discussion

### 3.1. Extraction Yields

For each extraction of red wine, seeds, and grape skins, an extraction yield has been calculated on the basis of the dried sample weight in gram units (Table 1).

Data show moderate extraction yields for grape skin extracts obtained by microwave, decoction, and soxhlet techniques. These values are higher than those found for organic phase 2 and organic phase 3, suggesting the development of further assays for the determination of antioxidant, antimicrobial and enzymatic inhibitory activities.

### 3.2. Phytochemical Composition

Total phenolic and flavonoid profiles for each extract were determined and reported in Table 2. The best phenolic content was found in wine-organic extract 2 (199.31 ± 7.21 mg GAE/g), which is three times higher than that of wine-aqueous extract (67.57 ± 0.16 mg GAE/g). This is probably due to the modification of the pH of the extraction phase (pH 7), which could promote the stabilization of the phenolic portion, improving the affinity for the organic phase. In addition, the dealcoholized wine extract and wine-organic extract 3, both obtained at pH 2, resulting in lower values of phenolic contents. Regarding the Montepulciano grape skin samples, the phenolic content is significantly lower than those of wine extracts. The values of grape skin extracts are quite similar to each other. Among them, soxhlet extraction furnished the best result. Wine extract 2 shows relevant data in terms of flavonoid content (22.76 ± 0.63 mg RE/g) compared to the other wine fractions, while grape skin flavonoids content in microwave-assisted decoction is the best result. This is not surprising since a high content in phenols has already been documented for other red wines, such as Zinfandel wine and Cabernet Sauvignon [33].

Phenolic Compounds are abundant in the wine-organic extract 2 and in the grape skin soxhlet extract; however, some of them could undergo metabolic degradation once in the organism suggesting a potential local application for the treatment of skin mouth wounds [34] or gastrointestinal illness [35].

### 3.3. Chromatographic Characterization of Phytochemicals

**Figure 2:**
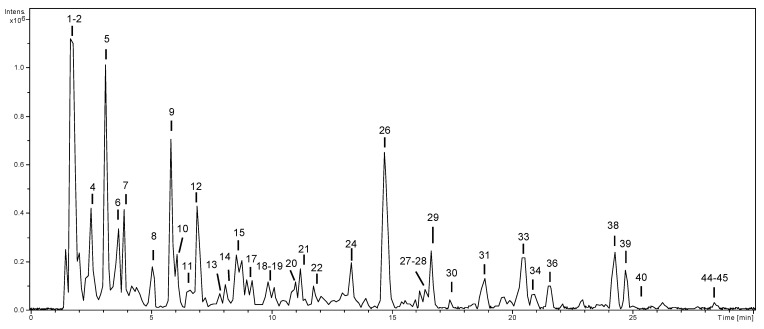
HPLC-ESI/MS^n^ base peak chromatograms (BPC) of dealcoholized wine.

Figure 2 is a base peak chromatogram representative of the first extract analyzed, e.g., dealcoholized wine.

Compounds **3** and **9**, with [M-H]^−^ at *m*/*z* 311 and fragment ions at *m*/*z* 179 and 135 (typical of caffeic acid), were tentatively characterized as caftaric acid isomers, previously described in wine samples [36] (MS data are in Table S1, see Supplementary Materials).

Compound **5** presented deprotonated molecular ion at *m*/*z* 169 and base peak at *m*/*z* 125, typical of gallic acid. Compound **25** was tentatively characterized as a derivative and Compound **26** as ethyl gallate [37].

Compound **6** was putatively characterized as 2-S-glutathionyl-caffeoyltartaric acid due to bibliographic information previously reported in wine [38].

Compounds **13** and **17**, with [M-H]^−^ at *m*/*z* 325, exhibited fragment ions at *m*/*z* 163 and 119 (coumaric acid). However, Compound **13** exhibited the direct loss of 162 Da (hexoside), whereas Compound **17** suffered losses of 60 and 90 Da (typical of C-glycosides). Therefore, Compound **13** was identified as coumaric acid-*O*-hexoside and **17** as coumaric acid-*C*-hexoside. Compound **15**, [M-H]^−^ at *m*/*z* 295, suffered the loss of tartaric acid (132 Da), yielding coumaric acid at *m*/*z* 163 (fragment ion at *m*/*z* 119), so it was identified as coutaric acid [39].

Compounds **16** and **18** presented deprotonated molecular ions at *m*/*z* 325 and, after the loss of 132 Da (pentoside), yielded ferulic acid at *m*/*z* 193 (fragment ions at *m*/*z* 149 and 134), so they were identified as ferulic acid-*O*-pentoside isomers. Compound **20** was identified as caffeic acid by comparison with an analytical standard. Compounds **14** and **22** exhibited the same fragmentation pattern, typical of (epi)catechin. They were characterized as catechin and epicatechin, respectively, by using an analytical standard of catechin. Compounds **11** and **19** were identified as procyanidin dimers due to the deprotonated molecular ion at *m*/*z* 577 and fragment ions at *m*/*z* 451, 425, 407, and 289 [40]. Compound **23** was identified as malvidin-*O*-glucoside based on the protonated molecular ion at *m*/*z* 493 and base peak at *m*/*z* 331 (malvidin). This Compound was predominant in grape skin and has been previously reported as one of the main pigments in these samples. This Compound was identified using the positive ion mode. Compound **42** was identified as myricetin due to the deprotonated molecular ion at *m*/*z* 317 and fragment ions at *m*/*z* 179 and 151. Compounds **27**, **28**, and **29** were characterized as glycosides (losses of 162 for hexoside and 176 Da for glucuronide), whereas Compounds **30**, **32**, and **35** as myricetin derivatives. Compound **33** suffered the neutral loss of 176 Da (glucuronide), and **37** and **39** lost 146 Da (deoxyhexoside) to yield the aglycone quercetin at *m*/*z* 301 (fragment ions at *m*/*z* 179 and 151), so they were characterized as quercetin-*O*-glucuronide and quercetin-*O*-deoxyhexoside isomers, respectively. In a similar way, Compounds **34**, **40**, and **44** were characterized as taxifolin (*m*/*z* 303, 285), syringetin (*m*/*z* 345, 330) and kaempferol (*m*/*z* 285, 257, and 255) glycosides. Compound **41** displayed the neutral loss of 308 Da (rutinoside), yielding isorhamnetin at *m*/*z* 315 (main fragment ion at *m*/*z* 300), so it was identified as isorhamnetin-*O*-rutinoside.

Compound **1** was characterized as the H_2_SO_4_ adduct of a disaccharide due to the ion at *m*/*z* 341 (disaccharide) and the fragmentation pattern of this ion [41]. Compounds **2** and **4**, with similar fragmentation patterns, were characterized as isocitric and citric acid, respectively, using an analytical standard of citric acid. Compound **8** was characterized as ethyl citrate according to bibliographic [42]. Compound **7**, with [M-H]^−^ at *m*/*z* 315, suffered the neutral loss of 162 Da (hexoside) to yield hydroxytyrosol at *m*/*z* 153 [38], so it was characterized as hydroxytyrosol hexoside. Compound **12** presented [M-H]^−^ at *m*/*z* 175 and fragment ions at *m*/*z* 157, 115, and 113. This fragmentation pattern has been previously reported for isopropylmalic acid [43]. Compounds **31** and **38** were characterized as trans-piceid and cis-piceid according to bibliographic data [38]. Compound **43** was tentatively characterized as laricitrin-3-*O*-rhamnose-7-*O*-trihydroxycinnamic acid, previously described in grape pomace [44]. A heatmap was constructed using the values of the areas (%) of each of the identified Compounds with respect to the total area in order to visualize the contribution of each of the Compounds to the extracts (Figure S1, see Supplementary Materials). Areas were calculated for each Compound using extracted ion chromatograms at the corresponding deprotonated molecular ion. In this way, it can be visually observed which Compounds are the most representative ones in each of the extracts. It could be observed that the profiles were very different depending on the extract. The main contributors to each wine extract were: (a) Dealcoholized wine: Compounds **1** (disaccharide), **5** (gallic acid), and **26** (ethyl gallate); (b) aqueous extract: Compounds **1** (disaccharide), **4** (citric acid) and **7** (hydroxytyrosol hexoside); (c) organic phase 2: **26** (ethyl gallate) and **33** + **39** (quercetin glycosides); (d) organic phase 3: Compound **12** (isopropylmalic acid). However, the dominant Compound in grape skin extracts was **23** (malvidin-*O*-glucoside). Hence, the bioactive properties were different depending on each of the extracts.

### 3.4. Trans-Resveratrol Quantification

**Figure 3:**
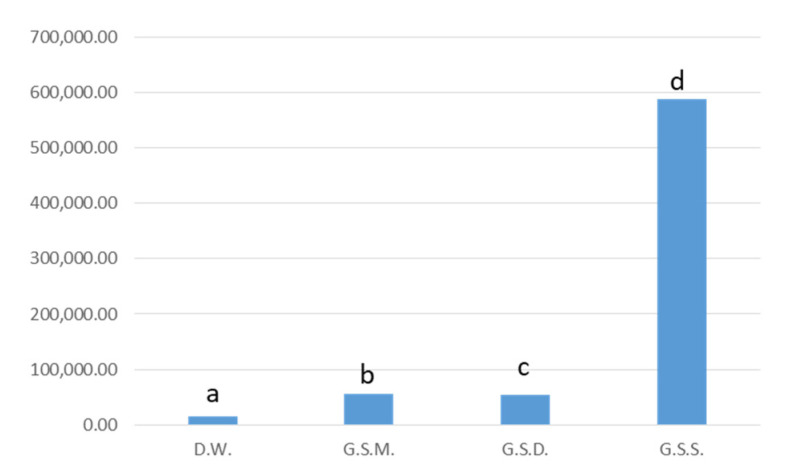
Resveratrol content in D.W. (dealcoholized wine), G.S.M. (grape skin microwave), G.S.D. (grape skin decoction), G.S.S. (grape skin soxhlet); the values are expressed in µg/g. Based on Tukey2019s assay at *p* < 0.05, different letters (a, b, c and d) indicated significant differences among the tested extracts (a: the highest value).

In this work, the *trans*-reveratrol content has been evaluated in the Montepulciano dealcoholized wine and in grape skin extracts obtained by soxhlet, decoction, and microwave-assisted extraction. As shown in the histogram (Figure 3), the content of resveratrol is comparable for grape skin decoction and microwave extract with a content of 750 µg/g and 575 µg/g, respectively, and was higher than that of dealcoholized wine (14.75 µg/g). These results can be explained considering that the resveratrol concentration in wine is influenced by the wine variety, grapes origin, and cultivar, ranging from 0.1 to 7 mg/L and from 0.7 to 6.5 mg/L as reviewed by Castaldo et al. [45].

The resveratrol content of red wine is lower than that found in the other extracts, which contain an enriched concentration of the selected Compounds.

### 3.5. Fatty Acid Composition

The fatty acid composition of grape seed oil was determined by using the GC-FID technique, and the results are shown in Table 3. Based on our results, C 18:2 ω6 (linoleic acid) was determined as the main fatty acid with a value of 69.29%. In accordance with our results, several researchers reported linoleic acid as the major fatty acid [46,47,48], but we observed a different level, which might be explained by diverse climatic conditions, geographical locations, and harvested times [49,50]. Besides linoleic acid, C18:1 ω9 (oleic acid) (18.57%) and C 16:0 (palmitic acid) (7.69%) are present in high levels when compared with other fatty acids. Clearly, the levels of total unsaturated fatty acids are higher than those of monounsaturated and saturated fatty acids. Total unsaturated fatty acids level was reached up to 80%. Essential fatty acids, namely linoleic and α-linolenic (C 18:3 ω3), are not synthesized by humans; therefore, they must be consumed by diet for maintaining normal physiological functions. As can be seen in Table 3, the tested oil contained a high level of essential oil (69.58%), and in this sense, it could be considered as a source of essential fatty acids.

### 3.6. Antioxidant Properties

As shown in Table 4, all wine extracts demonstrate appreciable antioxidant activity; wine-organic extract 2 exhibited suitable values in all assays, probably because it contains the best level of total phenolics. To provide a relationship between total bioactive Compounds (phenolics and flavonoids) and antioxidant effects, a correlation analysis was performed. The total amounts of the bioactive Compounds in the extracts strongly correlated with their antioxidant properties (Table 5). Pearson’s correlation values (R) were determined as 0.99 and 0.98 for DPPH and ABTS, respectively. In addition, the values were found to be 0.99 in the reducing power assays (CUPRAC and FRAP). In agreement with earlier studies, a linear correlation between total phenolic content and the antioxidant capacity was described in the literature [51]. The radical scavenging ability is due to the O-dihydroxylic structure common in polyphenol Compounds responsible for the stability of the radical form, also participating in electron delocalization [52,53]. Phenolic Compounds are known to be potent electron and hydrogen donors, and this phenomenon is important in understanding their role in the antioxidant assays [54]. However, a negative correlation between total bioactive Compounds and chelating metal ability (R: −0.71) was determined. This finding could be related to the presence of non-phenolic chelators in the tested extracts. Modest values were found by FRAP and DPPH for the aqueous wine extract (respectively 89.90 mgTEs/g for DPPH and 173.63 mgTEs/g for FRAP), while wine-organic extract 3 showed modest activity in ABTS and CUPRAC assay (respectively 144.83 mgTEs/g and 235.78 mgTEs/g). We also analyzed the antioxidant ability of grape skin, obtaining modest to low activity compared to the wine extracts, suggesting that the fermentation process could somehow influence the grape antioxidant properties [55,56,57]. The best values were provided by soxhlet extract and are, respectively, 101.25 mgTEs/g in ABTS and 142.71 mgTEs/g in CUPRAC. However, the oil showed no activity in DPPH and ABTS assays and low activity in the other antioxidant bioassays. The values reported by the decoction and microwave of grape skins are appreciable and similar to each other, confirming the polyphenol composition results. Data are quite low for grape seeds oil; however, it achieved a modest activity in the metal chelating assay. All results demonstrate the correlation between polyphenolic composition and the antioxidant effect of these natural molecules.

### 3.7. Enzyme Inhibition Assays

We further explored the inhibitory effects of wine, grape skins extracts, and seed oil against different enzymes involved in Alzheimer’s (AD) and diabetes mellitus (DM) diseases [58,59]. In this study, we evaluated the inhibitory activity of Montepulciano red wine and pomace extracts on these key enzymes. Our results reported in Table 6 show modest inhibition values of wine extracts against cholinesterases, which are important therapeutic targets involved in the modulation of neurotransmission in Alzheimer disease; wine-organic extract 2 and 3 exhibit the best values, respectively 8.95 ± 0.03 mgGALAEs/g and 8.89 ± 0.05 mgGALAEs/g in AChE inhibitory assay; these values are very close to those found in the BChE inhibition assay (8.11 ± 0.11 mgGALAE/g for wine-organic extract 2 and 7.71 ± 0.04 mgGALAE/g for wine-organic extract 3). We also studied the inhibitory activity of tyrosinase, the enzyme that converts tyrosine into L-DOPA and oxidizes L-DOPA to dopachrome [60,61]. Diverse studies reported the correlation between the inhibition of tyrosine and the polyphenolic content of red wine [62]. According to those, we found suitable inhibitory activity for wine-organic extract 2 with 151.30 ± 1.20 mgKAE/g, followed by the dealcoholized wine (139.28 ± 0.23 mgKAE/g) and the wine-organic extract 3 (137.89 ± 0.89 mgKAE/g). Our extracts show only modest inhibitory activity on amylase (0.77 ± 0.04 mmolACAE/g for organic extract 2), while a suitable activity on glucosidase was detected for dealcoholized wine (20.62 ± 0.23 mmolACAE/g) and aqueous extract (19.81 ± 0.03 mmolACAE/g); wine and grape skin extracts were inactive. Regarding grape skin extracts, the inhibition values are comparable to each other, while the seeds oil exhibits very low inhibitory activity in all the assays. Interestingly the skin extracts present suitable inhibitory activity on tyrosinase with 69.36 ± 0.23 mg KAE/g for decoction, 68.11 ± 0.44 mg KAE/g for decoction-MW, and 68.40 ± 0.16 mg KAE/g for soxhlet, albeit these values resulted lower than those found in red wine extracts, suggesting a possible involvement of fermentation process in the polyphenolic composition of red wine. Interestingly the extracts have suitable activity against tyrosinase, overcoming the activity of wine-aqueous extract. This underlines the direct correlation between polyphenolic composition and a possible anti-cancer activity that should be explored in the future.

### 3.8. In Vitro Assay

#### 3.8.1. Antimicrobial and Antifungal Activity Assays

In this work, we also evaluated the antimicrobial activity of Montepulciano red wine and grape pomace extracts against three bacterial strains and fungal culture. These data could be useful for the food industry and preservatives market [63,64]. The ability to inhibit microbial growth is attributed to the polyphenol component of wine, by others, to the aqueous fraction rich in organic acids. Several factors can affect the antimicrobial activity of polyphenolic Compounds: pH solution, solubility, and their ionization degree, solvent polarity, and their interactions with membrane proteins [65,66]. Some of these features, such as pH and solubility, are crucial because microbiological broths with specific properties are used (pH 7.4). The high antimicrobial and antifungal activity of polyphenols from grapes are often related to the very high concentration of Compounds used during assays [67,68]. In these experiments, the minimum inhibitory concentration for each extract sample was determined and reported in Table 7. Antimicrobial activity for all strains was higher than 256 µg/mL, and for *S. epidermidis* and *C. albicans* was in the range of 1024–256 µg/mL. In other cases, most extracts were active in a concentration higher than 1024 µg/mL. All wine extracts exhibit the same activity profile on *C. albicans*. The best value was found for a decoction extract that maintains antimicrobial activity in all the examined cultures (256 µg/mL for *C. albicans* and 512 µg/mL for bacterial strains). This result could be related to the composition of decoction extract, which has a suitable polyphenol content, moderate antioxidant activities, and an appreciable inhibition of tyrosinase. However, the soxhlet extract and wine-organic extracts are rich in polyphenols but do not have suitable antimicrobial activity, thus indicating that this is not the only determinant factor that contributes to the biological effect.

Industrial applications describe the interesting use of *Vitis vinifera* extracts alone or in combination with other natural plant extracts as antifungal preparations for the treatment of skin infections and in dermocosmetics [69,70,71].

#### 3.8.2. Anti-Inflammatory Activity on LPS-Stimulated Macrophage and Lipoxygenase (5-LOX) Inhibition

The anti-inflammatory activity of grape seed oil in human primary monocyte was recently documented by Millan-Linares et al. [61], and it is well known that curcumin and other plant-derived Compounds are able to reduce cytokine release from LPS-stimulated macrophages [72]. Macrophages are involved in the maintenance of the natural immune system and play immune regulatory functions. In our work, the effect of wine extracts on the viability of macrophages was studied at the indicated concentrations of 25, 50, and 100 µg/mL for 24 h (Table 8). Results show that macrophage viability was not significantly influenced by extracts at these concentrations and safely handled up to 100 μg/mL. LPS is the primary activator of macrophages that produce IL-6, TNF-α, and IL-1β [72]. Wine extracts induced a significant inhibitory effect on LPS-induced cytokine release at increasing concentration, reporting a maximal inhibitory effect at the dose of 100 μg/mL. Statistical analysis showed that W3 and W4 extracts were able to reduce cytokine release at the starting concentration of 25 µg/mL, being the most effective of the series. This could be due to the well-described anti-inflammatory properties of resveratrol in red wine [73], which was quantified in dealcoholized wine and grape skin extracts (Section 3.4) as well as procyanidin dimer in dealcoholized wine and malvidin-*O*-glucoside in grape skin extracts belonging to the big family of antocyanines (see Supplementary Materials). In particular malvidin-*O*-glucoside accounts for 49.17%, 32.52%, and 49.88% of the relative peak area of grape skin decoction, soxhlet, and microwave extracts, respectively [74]. Moderate consumption of wine reduced the incidence of coronary heart diseases because alcohol decreases platelet aggregation into the endothelial surface of the arteries, blood coagulation, and thrombus [75]. Inhibition of cyclooxygenase-1 (COX-1) resulted in being a valuable approach to prevent cardiovascular diseases, as it catalyzes the formation of thromboxanes and eicosanoids, prompting aggregation and vasoconstriction. Phenolic Compounds such as resveratrol are reported as inhibitors of COX-1 [75,76]. In addition, the enzyme 5-lipoxygenase (5-LOX) mediates the formation of eicosanoids in a second biosynthetic pathway; the final product, leukotriene B4 (LTB4), is involved in inflammatory diseases, including atherosclerosis [76]. Quercetin inhibits 5-LOX activity, but some recent works reported a strong activation of COX-1 and COX-2 by the latter Compound and myricetin, suggesting an increasing eicosanoids formation by wine [76,77]. In addition, catechins (EGCG, EGC, and EC) in green tea influence the IL-1β signaling pathway regulating the expression of pro-inflammatory mediators (IL-6, IL-8) and COX-2 in primary human rheumatoid arthritis synovial fibroblasts (RASFs) [75,76,77,78]. EGCG significantly inhibited constitutive COX-2 mRNA and protein overexpression and down-regulated the ERK1/2 and Akt pathways in colon cancer cells [79]. In this work, wine extracts showed a dose-dependent inhibition of 5-LOX with 30–50% and 100% at the tested concentrations of 10 and 100 μg/mL, respectively (Table 9). No differences were observed among the extracts in any of the doses tested, indicating that all of them possess the same inhibitory action on 5-LOX activity.

## 4. Conclusions

In this study, polyphenolic and flavonoid compositions, antioxidant activity, enzyme inhibition, and antimicrobial activity of wine and grape pomace (skin/pulp and seeds) extracts of Montepulciano d’Abruzzo were analyzed. In wine extracts, phenolic and flavonoid contents were higher than those found in grape skins. Wine-organic 2 phase obtained from EtOAc extraction at pH 7 has the best content of phenolic Compounds and phenolic acids. In the antioxidant assays, this extract achieved very suitable values in each test, particularly in the CUPRAC assay with 1036 mg TE/g. In addition, the wine-organic extract 2 also performed well in enzyme inhibition assay, especially on α-tyrosinase. Modest results were collected by anti-inflammatory activity on LPS-stimulated macrophage for wine extracts, inducing a significant inhibitory effect on LPS-induced cytokine release. In this regard, an increase in cytokine concentration and a maximal inhibitory effect at the dose of 100 μg/mL were observed for all the wine extracts. Overall our analysis highlights the phytochemical composition and biological profile as an antioxidant, enzymatic inhibitor, and antimicrobial agent of grape pomace extracts from Montepulciano d’Abruzzo compared to Montepulciano d’Abruzzo red wine. The soxhlet extract of grape skin furnished the best result in polyphenolic Compounds, antioxidant activity, and enzyme inhibition, while data from grape seed oil are not significant, with the only exception for PUFA and EFA contents. The increasing demand for natural Compounds for sustainable agriculture suggests the need to develop a suitable method to reuse and valorize the grape pomace composition in bioactive components, limiting its use as compost while pushing on the other side, its involvement in profitable applications such as food processing, functional foods, cosmetics, and supplements. This work represents a starting point to design healthy ingredients and functional foods from grape pomace waste of red wine production in the Abruzzo region.

## Figures and Tables

**Table 1:** Extraction yields calculated as % *w*/*w* for wine-aqueous extract, organic phase 2, organic phase 3, grape skin MW, decoction, and soxhlet and seeds oil.

Extracts	Extract Yield (% *w*/*w*) ± S.D.
Wine-aqueous extract	46.9 ± 0.1
Organic phase 2	6.5 ± 0.2
Organic phase 3	2.5 ± 0.2
Grape skin MW	43.1 ± 0.1
Grape skin decoction	26.5 ± 0.1
Grape skin soxhlet	43.3 ± 0.1
Seeds oil	9.9 ± 0.3

Values expressed are means ± S.D. of three parallel measurements.

**Table 2:** Total phenolic, flavonoid contents of the tested extracts.

Samples	Total Phenolic Content (mgGAE/g)	Total Flavonoid Content (mgRE/g)
Dealcoholized wine	51.86 ± 0.80 ^c^	2.27 ± 0.04 ^d^
Wine-aqueous extract	67.57 ± 0.16 ^b^	0.93 ± 0.09 ^e^
Wine-organic extract 2	199.31 ± 7.21 ^a^	22.76 ± 0.63 ^a^
Wine-organic extract 3	60.78 ± 0.52 ^b^	3.41 ± 0.03 ^c^
Decoction	29.88 ± 0.37 ^d^	6.81 ± 0.08 ^b^
Decoction-MW	27.04 ± 0.13 ^d^	6.86 ± 0.14 ^b^
Soxhlet	31.56 ± 0.27 ^d^	6.42 ± 0.05 ^b^
Oil	12.03 ± 0.07 ^e^	0.09 ± 0.01 ^f^

Values expressed are means ± S.D. of three parallel measurements. GAE: Gallic acid equivalent; RE: Rutin equivalent. Different letters in the same column indicate significant differences in the tested samples (*p* < 0.05).

**Table 3:** Fatty acid composition of grape seed oil (%).

Fatty Acids (Common Names)	Oil
C 14:0 (myristic acid)	0.06 ± 0.01
C 15:0 (pentadesilik acid)	0.01 ± 0.01
C 16:0 (palmitic acid)	7.69 ± 0.02
C 17:0 (margaric acid)	0.09 ± 0.01
C 18:0 (stearic acid)	3.75 ± 0.04
ΣSFA	11.58 ± 0.07
C 14:1 ω5 (myristoleic acid)	0.01 ± 0.01
C 15:1 ω5 (pentadecanoic acid)	0.01 ± 0.01
C 16:1 ω7 (palmitoleic acid)	0.03 ± 0.01
C 17:1 ω8 (margarioleic acid)	0.08 ± 0.01
C 18:1 ω9 (oleic acid)	18.57 ± 0.01
ΣMUFA	18.70 ± 0.01
C 18:2 ω6 (linoleic acid)	69.29 ± 0.04
C 18:3 ω6 (γ-linolenic acid)	0.16 ± 0.02
C 18:3 ω3 (α-linolenic acid)	0.30 ± 0.02
ΣPUFA	69.74 ± 0.08
ΣUFA	88.43 ± 0.08
ΣEFA	69.58 ± 0.01

Values are reported as mean ± SD of three GC replications. SFA: Saturated fatty acids; MUFA: Monounsaturated fatty acids; PUFA: Polyunsaturated fatty acids; UFA: Unsaturated fatty acids; EFA: Essential fatty acids.

**Table 4:** Antioxidant properties of wine, grape skin extracts, and oil.

Samples	DPPH (mg TE/g)	ABTS (mg TE/g)	CUPRAC (mg TE/g)	FRAP (mg TE/g)	Metal Chelating (mg EDTAE/g)	PPBD (mmol TE/g)
Dealcoholized wine	76.28 ± 0.73 ^c^	136.65 ± 0.41 ^c^	192.73 ± 0.45 ^d^	134.03 ± 0.40 ^c^	1.25 ± 0.30 ^e^	1.11 ± 0.06^c^
Wine-aqueous extract	89.90 ± 1.15 ^b^	115.93 ± 0.40 ^d^	210.44 ± 0.83 ^c^	173.63 ± 1.91 ^b^	na	1.52 ± 0.11 ^b^
Wine-organic extract 2	417.02 ± 6.83 ^a^	733.41 ± 4.74 ^a^	1036.98 ± 14.01 ^a^	885.71 ± 20.02 ^a^	na	3.74 ± 0.19 ^a^
Wine-organic extract 3	85.39 ± 1.93 ^b^	144.83 ± 1.25 ^b^	235.78 ± 2.64 ^b^	164.29 ± 0.59 ^b^	2.06 ± 0.10 ^d^	1.27 ± 0.24 ^bc^
Decoction	47.22 ± 0.10 ^d^	99.04 ± 0.95 ^e^	127.24 ± 2.50 ^f^	71.59 ± 0.32 ^d^	5.36 ± 0.16 ^b^	1.04 ± 0.06 ^c^
Decoction-MW	44.80 ± 0.31 ^d^	91.15 ± 1.03 ^f^	115.36 ± 0.59 ^f^	61.27 ± 0.17 ^e^	4.70 ± 0.35 ^c^	0.94 ± 0.01 ^c^
Soxhlet	48.37 ± 0.84 ^d^	101.25 ± 0.78 ^e^	142.71 ± 1.32 ^e^	78.16 ± 0.29 ^d^	6.12 ± 0.04 ^a^	1.11 ± 0.04 ^c^
Oil	na	na	37.37±0.70 ^g^	14.28±0.16 ^f^	5.54±0.32 ^a,b^	0.51±0.13 ^d^

Values are reported as mean ± SD. of three parallel experiments. TE: Trolox equivalents; EDTAE: EDTA equivalent. na: not active. PPBD: Phosphomolybdenum assay. Different letters in the same column indicate significant differences in the tested samples (*p* < 0.05).

**Table 5:** Pearson’s correlation (R) values between total phenolic, flavonoid content, and antioxidant assays.

	TPC	TFC	DPPH	ABTS	CUPRAC	FRAP	MCA	PBD
TPC	1.00	0.85	0.99	0.98	0.99	0.99	−0.71	0.99
TFC	0.85	1.00	0.90	0.93	0.91	0.90	−0.28	0.89
DPPH	0.99	0.90	1.00	1.00	1.00	1.00	−0.64	0.99
ABTS	0.98	0.93	1.00	1.00	1.00	0.99	−0.58	0.99
CUPRAC	0.99	0.91	1.00	1.00	1.00	1.00	−0.61	0.99
FRAP	0.99	0.90	1.00	0.99	1.00	1.00	−0.63	0.99
MCA	−0.71	−0.28	−0.64	−0.58	−0.61	−0.63	1.00	−0.65
PBD	0.99	0.89	0.99	0.99	0.99	0.99	−0.65	1.00

CA: Metal chelating assay; PBD: Phosphomolybdenum.

**Table 6:** Enzyme inhibitory properties of wine extracts.

Samples	AChE Inhibition (mgGALAE/g)	BChE Inhibition (mgGALAE/g)	Tyrosinase Inhibition (mgKAE/g)	Amylase Inhibition (mmolACAE/g)	Glucosidase Inhibition (mmolACAE/g)
Dealcoholized wine	8.62 ± 0.01 ^b^	4.16 ± 0.19 ^b,c^	139.28 ± 0.23 ^b^	0.37 ± 0.01 ^c^	20.62 ± 0.23 ^a^
Wine-aqueous extract	8.27 ± 0.02 ^c^	4.66 ± 0.17 ^b^	7.64 ± 0.31 ^e^	0.09 ± 0.01 ^d^	19.81 ± 0.03 ^b^
Wine-organic extract 2	8.89 ± 0.05 ^a^	8.11 ± 0.11 ^a^	151.30 ± 1.20 ^a^	0.77 ± 0.04 ^a^	na
Wine-organic extract 3	8.95 ± 0.03 ^a^	7.71 ± 0.04 ^a^	137.89 ± 0.89 ^b^	0.76 ± 0.02 ^a^	na
Decoction	2.58 ± 0.03 ^d^	3.25 ± 0.45 ^d^	69.36 ± 0.23 ^c^	0.46 ± 0.02 ^b^	na
Decoction-MW	2.52 ± 0.02 ^d^	3.93 ± 0.22 ^c^	68.11 ± 0.44 ^c^	0.45 ± 0.01 ^b^	na
Soxhlet	2.53 ± 0.03 ^d^	3.16 ± 0.11 ^d^	68.40 ± 0.16 ^c^	0.46 ± 0.02 ^b^	na
Oil	0.98 ± 0.04 ^e^	2.29 ± 0.07 ^e^	24.86 ± 0.84 ^d^	0.35 ± 0.02 ^c^	na

Values expressed are means ± S.D. of three parallel measurements; AChE: acetylcholinesterase; BChE: butyrylcholinesterase; GALAE: Galantamine equivalent; KAE: Kojic acid equivalent; ACAE: Acarbose equivalent. na: not active. Different letters in the same column indicate significant differences in the tested samples (*p* < 0.05).

**Table 7:** Results of antimicrobial assay-minimum inhibitory concentration (MIC).

	*Staphylococcus aureus* ATCC 6538	*Staphylococcus epidermidis* PCM 2532	*Escherichia coli* ATCC 25922	*Candida albicans* ATCC 10231
Dealcoholized red wine	>1024 µg/mL	1024 µg/mL	>1024 µg/mL	512 µg/mL
Aqueous extract	>1024 µg/mL	>1024 µg/mL	>1024 µg/mL	512 µg/mL
Organic extract 2	>1024 µg/mL	1024 µg/mL	>1024 µg/mL	512 µg/mL
Organic extract 3	>1024 µg/mL	1024 µg/mL	>1024 µg/mL	512 µg/mL
Decoction	512 µg/mL	512 µg/mL	512 µg/mL	256 µg/mL
Decoction-MW	1024 µg/mL	512 µg/mL	1024 µg/mL	1024 µg/mL
Soxhlet	>1024 µg/mL	1024 µg/mL	1024 µg/mL	1024 µg/mL
Oil	>1024 µg/mL	1024 µg/mL	>1024 µg/mL	1024 µg/mL

Used extracts concentrations: 1024 µg/mL–2 µg/mL. Activity of extracts was examined using broth microdilution method in Mueller Hinton II Broth for bacteria and RPMI 1640 for fungus.

**Table 8:** Effects induced by extracts on cell viability and LPS-induced cytokine release (IL-6, TNF-α, and IL-1β) in macrophages. Macrophages were exposed for 24 h to 25, 50, and 100 µg/mL of extracts.

Sample (µg/mL)	Cell Viability-MTT (%) (Mean ± SEM)	IL-6 Release (%) (Mean ± SEM)	TNF-α Release (%) (Mean ± SEM)	IL-1β Release (%) (Mean ± SEM)
Dealcoholized wine (25)	94.0 ± 6.0	72.0 ± 7.2 ^a^	88.7 ± 5.4	85.3 ± 7.6
Dealcoholized wine (50)	86.5 ± 5.8	40.0 ± 4.3 ^d^	86.5 ± 6.1	58.5 ± 9.5 ^b^
Dealcoholized wine (100)	95.1 ± 6.5	26.5 ± 2.2 ^d^	49.8 ± 6.4 ^c^	35.0 ± 2.3 ^d^
Wine-aqueous extract (25)	99.6 ± 3.3	75.2 ± 7.1	89.5 ± 5.2	80.5 ± 7.6
Wine-aqueous extract (50)	100.0 ± 10	36.3 ± 5.7 ^d^	81.7 ± 4.7	43.5 ± 5.3 ^d^
Wine-aqueous extract (100)	74.9 ± 2.1	27.5 ± 4.6 ^d^	46.2 ± 4.3 ^d^	39.0 ± 3.1 ^d^
Wine-organic extract 2 (25)	98.3 ± 3.8	58.7 ± 9.3 ^b^	73.5 ± 10.3	55.1 ± 7.2 ^a^
Wine-organic extract 2 (50)	100.0 ± 12.3	21.3 ± 3.3 ^d^	49.5 ± 6.6 ^b^	34.8 ± 4.4 ^b^
Wine-organic extract 2 (100)	91.7 ± 4.8	10.7 ± 1.1 ^d^	29.0 ± 3.0 ^d^	19.0 ± 2.8 ^b^
Wine-organic extract 3 (25)	100.0 ± 6.4	67.7 ± 8.7 ^a^	81.5 ± 4.9	65.6 ± 8.2 ^a^
Wine-organic extract 3 (50)	100.0 ± 6.5	44.2 ± 6.0 ^c^	52.0 ± 5.4 ^c^	35.6 ± 4.4 ^d^
Wine-organic extract 3 (100)	93.9 ± 8.0	18.0 ± 2.0 ^d^	28.2 ± 3.7 ^d^	26.6 ± 3.8 ^d^
Decoction (25)	92.8 ± 2.8	92.3 ± 3.2	96.4 ± 3.6	91.7 ± 5.2
Decoction (50)	96.5 ± 2.4	90.1 ± 5.6	91.6 ± 6.2	90.8 ± 5.2
Decoction (100)	92.5 ± 3.4	86.2 ± 5.7	74.2 ± 6.8 ^b^	82.3 ± 6.7
Decoction-MW (25)	94.2 ± 3.4	95.6 ± 1.9	93.5 ± 3.8	95.7 ± 2.7
Decoction-MW (50)	91.4 ± 4.6	96.1 ± 1.6	92.3 ± 4.5	97.8 ± 0.9
Decoction-MW (100)	94.2 ± 2.9	89.9 ± 5.9	96.0 ± 2.6	80.7 ± 8.1 ^a^
Soxhlet (25)	98.3 ± 1.2	96.2 ± 3.5	96.4 ± 2.4	92.5 ± 3.6
Soxhlet (50)	97.4 ± 1.6	88.6 ± 5.2	85.4 ± 4.7	91.2 ± 4.5
Soxhlet (100)	94.2 ± 2.9	75.2 ± 6.4 ^b^	76.0 ± 4.2 ^b^	74.1 ± 6.7 ^b^
Oil (25)	92.2 ± 4.2	97.8 ± 1.9	98.7 ± 1.1	95.8 ± 2.2
Oil (50)	96.7 ± 2.1	96.8 ± 2.1	94.3 ± 3.9	93.1 ± 2.1
Oil (100)	94.3 ± 3.5	96.4 ± 2.3	97.7 ± 2.0	96.7 ± 3.7

^a^ is for *p* < 0.05, ^b^ is for *p* < 0.01, ^c^ is for *p* < 0.001 and ^d^ is for *p* < 0.0001 vs. LPS. Results were obtained from three separate experiments performed in duplicate and reported as mean ± SEM. Data were analyzed using one-way ANOVA followed by Tukey’s post-test.

**Table 9:** Inhibitory effects of extracts on 5-LOX activity.

Sample (µg/mL)	Inhibition %
Dealcoholized wine (10)	35 ± 0.7
Dealcoholized wine (100)	100 ± 1.3
Wine-aqueous extract (10)	45 ± 1.3
Wine-aqueous extract (100)	100 ± 0.6
Wine-organic extract 2 (10)	50 ± 4.4
Wine-organic extract 2 (100)	100 ± 1.3
Wine-organic extract 3 (10)	50 ± 5.0
Wine-organic extract 3 (100)	100 ± 1.7
Decoction (10)	34 ± 5.3
Decoction (100)	47 ± 6.3
Decoction-MW (10)	23 ± 2.1
Decoction-MW (100)	35 ± 4.5
Soxhlet (10)	28 ± 3.8
Soxhlet (100)	44 ± 7.5
Oil (10)	15 ± 0.7
Oil (100)	24 ± 3.2

Data were obtained from five separate experiments performed in duplicate and reported as mean ± SEM.

## Data Availability

The data presented in this study are available in the article and Supplementary Materials.

## Supplementary Materials

The following are available online at https://www.mdpi.com/article/10.3390/antiox10111704/s1, Reagents and standard, HPLC conditions, anti-inflammatory assay. Table S1: Characterization of phytochemicals in extracts of wine by HPLC–ESI–MS^n^, Figure S1: Relative peak areas (%) and heat map obtained by HPLC-ESI-MS^n^ analysis of wine extracts, Figure S2: Calibration curve obtained by linear regression of areas against the resveratrol concentration (µg).
