# Supplementary material for: Phenolic Analysis and In Vitro Biological Activity of Red Wine, Pomace and Grape Seeds Oil Derived from Vitis vinifera L. cv. Montepulciano d’Abruzzo
Source: Antioxidants (Basel). 2021 Oct 27;10(11):1704. doi: 10.3390/antiox10111704 (PMC8615145; doi:10.3390/antiox10111704)
Supplement: Supplementary file 1 [file antioxidants-10-01704-s001.zip › antioxidants-1434577-supplementary.pdf]

Supporting information

Phenolic analysis and *in vitro* biological activity of red wine, pomace and grape seeds oil derived from *Vitis vinifera* L. cv. Montepulciano d'Abruzzo

Adriano Mollica,[1] Giuseppe Scioli,[1] Alice Della Valle,[1] Angelo Cichelli,[2] Ettore Novellino,[3] Marta Bauer,[4] Wojciech Kamysz,[4] Eulogio J. Llorent-Martínez,[5] Maria Luisa Fernández-de Córdova,[5] R. Castillo-López,[5] Gunes Ak,[6] Gokhan Zengin,[6] Stefano Pieretti,[7] Azzurra Stefanucci[1,*]

[1]Department of Pharmacy, G. d'Annunzio University of Chieti-Pescara, 66100 Chieti, Italy; giuseppe.scioli@unich.it (G.S.); alice.dellavalle@unich.it (A.D.V.); a.mollica@unich.it (A.M.); a.stefanucci@unich.it (A.S.)

[2]Department of Medical, Oral and Biotechnological Sciences, "G. d'Annunzio" University Chieti-Pescara, Via dei Vestini 31, 66100 Chieti, Italy; angelo.cichelli@unich.it

[3] NGN-Healthcare Avellino Italy; ettorenovellino09@gmail.com

[4]Department of Inorganic Chemistry, Faculty of Pharmacy, Medical University of Gdànsk, 80-416 Gdànsk, Poland; marta.bauer@gumed.edu.pl (M.B.); wojciech.kamysz@gumed.edu.pl (W.K.)

[5]University of Jaén, Faculty of Experimental Sciences, Department of Physical and Analytical Chemistry, E-23071 Jaén, Spain; ellorent@ujaen.es (E.J.L.M.); mferna@ujaen.es (M.L.F.C.); rcl00041@red.ujaen.es (R.C.L.)

[6]Department of Biology, Science Faculty, Selcuk University, Konya 42130, Turkey; gokhanzengin@selcuk.edu.tr (G.Z.); akguneselcuk@gmail.com (G.A.)

[7]National Center for Drug Research and Evaluation, Italian National Institute of Health, Viale Regina Elena 299, 00161 Rome, Italy; stefano.pieretti@iss.it

*Correspondence: a.stefanucci@unich.it; 08713554904

1. Chromatographic analyses of extracts

Chromatographic analyses were performed with an Agilent Series 1100 with a G1315B diode array detector (Agilent Technologies, Santa Clara, CA, USA), a re-versed-phase Luna Omega Polar C18 analytical column (150 x 3.0 mm; 5 µm particle size; Phenomenex, Torrance, CA, USA) and a Polar C18 Security Guard cartridge of 4 x 3.0 mm (Phenomenex). The mobile phases consisted of water + formic acid 0.1 % v/v (eluent A) and acetonitrile (eluent B). The gradient elution was: 10-25% B in 0-25 min, 25% B in 25-30 min, 25-50% B in 30-40 min, 50-100% B in 40-42 min, 100% in 42-47 min. Then, eluent B was returned to 10% with a 7 min stabilization time. The flow rate was 0.4 mL min^{-1}.

1.2 Identification of individual phenolic compounds by HPLC-ESI-MS method

The HPLC system was connected to an ion trap mass spectrometer (Esquire 6000, Bruker Daltonics, Billerica, MA, USA) equipped with an electrospray interface. The scan range was at m/z 100-1200 with a speed of 13,000 Da/s. The ESI conditions were: drying gas (N_2) flow rate and temperature, 10 mL/min and 365 ºC; nebulizer gas (N_2) pressure, 50 psi; capillary voltage, 4500 V; capillary exit voltage, -117.3 V. We used the auto MS^n mode for the acquisition of MS^n data, with isolation width of 4.0 m/z, and fragmentation amplitude of 0.6 V (MS^n up to MS^4).

2. Reagents and standards

For sample preparation and chromatographic analysis, deionized water with ≥ 18 MΩ•cm resistivity purified with a Milli-Q system (Millipore, Bedford, USA) was used. All solvents and solutions were filtered through a 0.45-µm polytetrafluoroethylene (PTFE) filter from Supelco (Bellefonte, PA, USA) before use. 5,5-Dithio-bis(2-nitrobenzoic) acid) (DTMB), AChE (electric eel AChE, Type-VI-S, EC 3.1.1.7), BChE (horse serum BChE, EC 3.1.1.8), acetylthiocholine iodide (ATCI), butyrylthiocholine chloride (BTCl), galantamine hydrobromide (from Lycoris sp., ≥94%), α-amylase (porcine pancreas, EC 3.2.1.1), α-glucosidase (from Saccharomyces cerevisiae, EC 3.2.1.20), 4-nitrophenyl-α-D-glucopyranoside (PNPG), tyrosinase (from mushroom, EC 1.14.18.1), L-2,3-dihydroxyphenylalanine (L-DOPA), kojic acid (≥99%) and acarbose (≥95%) were purchased from Sigma-Aldrich (St. Louis, MO, USA. Analytical standards of gallic acid, rutin, caffeic acid and 6-hydroxy-2,5,7,8-tetramethylchroman-2-carboxylic acid (Trolox) (purity ≥95%) were acquired from Sigma-Aldrich (Milan, Italy).

3. HPLC Analysis Conditions

Agilent Series 1100 with a G1315B diode array detector (Agilent Technologies, Santa Clara, CA, USA) with a reversed-phase Luna Omega Polar C_{18} analytical column (150 x 3.0 mm; 5 µm particle size; Phenomenex, Torrance, CA, USA) and a Polar C_{18} Security Guard cartridge of 4 x 3.0 mm (Phenomenex) were used. The following conditions were applied: mobile phases = water + formic acid 0.1 % v/v (eluent A) and acetonitrile (eluent B); gradient elution = 10-25% B in 0-25 min, 25% B in 25-30 min, 25-50% B in 30-40 min, 50-100% B in 40-42 min, 100% in 42-47 min.; flow rate = 0.4 mL min^{-1}.

The HPLC system was connected to an ion trap mass spectrometer (Esquire 6000, Bruker Daltonics, Billerica, MA, USA) equipped with an electrospray interface. The scan range was at m/z 100–1200 with a speed of 13,000 Da/s. The ESI conditions were: drying gas (N_2) flow rate and temperature, 10 L/min and 365 ºC; nebulizer gas (N_2) pressure, 50 psi; capillary voltage, 4500 V; capillary exit voltage, -117.3 V. We used the auto MS^n mode for the acquisition of MS^n data, with isolation width of 4.0 m/z, and fragmentation amplitude of 0.6 V (MS^n up to MS^4).

4. Assays for Total Phenolic and Flavonoid Contents

The total phenolic content was determined by employing the methods given in the literature with some modification. Sample solution (0.25 mL) was mixed with diluted Folin–Ciocalteu reagent (1 mL, 1:9, v/v) and shaken vigorously. After 3 min, Na_2CO_3 solution (0.75 mL, 1%) was added and the sample absorbance was read at 760 nm after a 2 h incubation at room temperature. The total phenolic content was expressed as milligrams of gallic acid equivalents (mg GAE/g extract)[1]. Calibration curve was: absorbance=0.2159 (µg gallic acid), R^2=0.9962

The total flavonoid content was determined using the $AlCl_3$ method. Briefly, sample solution (1 mL) was mixed with the same volume of aluminum trichloride (2%) in methanol. Similarly, a blank was prepared by adding sample solution (1 mL) to methanol (1 mL) without $AlCl_3$. The sample and blank absorbances were read at 415 nm after a 10 min incubation at room temperature. The absorbance of the blank was subtracted from that of the sample. Rutin was used as a reference standard and the total flavonoid content was expressed as milligrams of rutin equivalents (mg RE/g extract) [1]. Calibration curve was: absorbance=0.130 (µg rutin)+0.0512, R^2=0.9934

5. Determination of Antioxidant and Enzyme Inhibitory Effects

Antioxidant (DPPH and ABTS radical scavenging, reducing power (CUPRAC and FRAP), phosphomolybdenum and metal chelating (ferrozine method)) and enzyme inhibitory activities (cholinesterase (Elmann's method), tyrosinase (dopachrome method), α-amylase (iodine/potassium iodide method), α -glucosidase (chromogenic PNPG method) and pancreatic lipase (*p*-nitrophenyl butyrate (p-NPB) method) were determined using the methods previously described by Uysal *et al.* [1] and Grochowski *et al.* [2]

For the DPPH (1,1-diphenyl-2-picrylhydrazyl) radical scavenging assay: Sample solution was added to 4 mL of a 0.004% methanol solution of DPPH. The sample absorbance was read at 517 nm after a 30 min incubation at room temperature in the dark. DPPH radical scavenging activity was expressed as milligrams of trolox equivalents (mg TE/g extract).

For ABTS (2,2′-azino-bis(3-ethylbenzothiazoline) 6-sulfonic acid) radical scavenging assay: Briefly, ABTS+ was produced directly by reacting 7 mM ABTS solution with 2.45 mM potassium persulfate and allowing the mixture to stand for 12–16 in the dark at room temperature. Prior to beginning the assay, ABTS solution was diluted with methanol to an absorbance of 0.700 ± 0.02 at 734 nm. Sample solution was added to ABTS

solution (2 mL) and mixed. The sample absorbance was read at 734 nm after a 30 min incubation at room temperature. The ABTS radical scavenging activity was expressed as milligrams of trolox equivalents (mg TE/g extract).

For CUPRAC (cupric ion reducing activity) activity assay: Sample solution was added to premixed reaction mixture containing $CuCl_2$ (1 mL, 10 mM), neocuproine (1 mL, 7.5 mM) and NH_4Ac buffer (1 mL, 1 M, pH 7.0). Similarly, a blank was prepared by adding sample solution (0.5 mL) to premixed reaction mixture (3 mL) without $CuCl_2$. Then, the sample and blank absorbances were read at 450 nm after a 30 min incubation at room temperature. The absorbance of the blank was subtracted from that of the sample. CUPRAC activity was expressed as milligrams of trolox equivalents (mg TE/g extract).

For FRAP (ferric reducing antioxidant power) activity assay: Sample solution was added to premixed FRAP reagent (2 mL) containing acetate buffer (0.3 M, pH 3.6), 2,4,6-tris(2-pyridyl)-S-triazine (TPTZ) (10 mM) in 40 mM HCl and ferric chloride (20 mM) in a ratio of 10:1:1 (v/v/v). Then, the sample absorbance was read at 593 nm after a 30 min incubation at room temperature. FRAP activity was expressed as milligrams of trolox equivalents (mg TE/g extract).

For phosphomolybdenum method: Sample solution was combined with 3 mL of reagent solution (0.6 M sulfuric acid, 28 mM sodium phosphate and 4 mM ammonium molybdate). The sample absorbance was read at 695 nm after a 90 min incubation at 95 °C. The total antioxidant capacity was expressed as millimoles of trolox equivalents (mmol TE/g extract).

For metal chelating activity assay: Briefly, sample solution was added to $FeCl_2$ solution (0.05 mL, 2 mM). The reaction was initiated by the addition of 5 mM ferrozine (0.2 mL). Similarly, a blank was prepared by adding sample solution (2 mL) to $FeCl_2$ solution (0.05 mL, 2 mM) and water (0.2 mL) without ferrozine. Then, the sample and blank absorbances were read at 562 nm after 10 min incubation at room temperature. The absorbance of the blank was sub-tracted from that of the sample. The metal chelating activity was expressed as milligrams of EDTA (disodium edetate) equivalents (mg EDTAE/g extract).

For Cholinesterase (ChE) inhibitory activity assay: Sample solution (was mixed with DTNB (5,5-dithio-bis(2-nitrobenzoic) acid, Sigma, St. Louis, MO, USA) (125 µL) and AChE (acetylcholines-terase (Electric ell acetylcholinesterase, Type-VI-S, EC 3.1.1.7,Sigma)), or BChE (butyrylcholinesterase (horse serum butyrylcholinesterase, EC 3.1.1.8, Sigma)) solution (25 µL) in Tris–HCl buffer (pH 8.0) in a 96-well microplate and incubated for 15 min at 25 °C. The reaction was then initiated with the addition of acetylthiocholine iodide (ATCI, Sigma) or butyrylthiocholine chloride (BTCl, Sigma) (25 µL). Similarly, a blank was prepared by adding sample solution to all reaction reagents without enzyme (AChE or BChE) solution. The sample and blank absorbances were read at 405 nm after 10 min incubation at 25 °C. The absorbance of the blank was subtracted from that of the sample and the cholinesterase inhibitory activity was expressed as galanthamine equivalents (mgGALAE/g extract).

For Tyrosinase inhibitory activity assay: Sample solution was mixed with tyrosinase solution (40 µL, Sigma) and phosphate buffer (100 µL, pH 6.8) in a 96-well microplate and incubated for 15 min at 25 °C. The reaction was then initiated with the addition of L-DOPA (40 µL, Sigma). Similarly, a blank was prepared by adding sample solution to all reaction reagents without enzyme (tyrosinase) solution. The sample and blank absorbances were read at 492 nm after a 10 min incubation at 25 °C. The absorbance of the blank was subtracted from that of the sample and the tyrosinase inhibitory activity was expressed as kojic acid equivalents (mgKAE/g extract).

For α-amylase inhibitory activity assay: Sample solution was mixed with α-amylase solution (ex-porcine pancreas, EC 3.2.1.1, Sigma) (50 µL) in phosphate buffer (pH 6.9 with 6 mM sodium chloride) in a 96-well microplate and incubated for 10 min at 37 °C. After pre-incubation, the reaction was initiated with the addition of starch solution (50 µL, 0.05%). Similarly, a blank was prepared by adding sample solution to all reaction reagents without enzyme (α-amylase) solution. The reaction mixture was incubated 10 min at 37 °C. The reaction was then stopped with the addition of HCl (25 µL, 1 M). This was followed by addition of the iodine-potassium iodide solution (100 µL). The sample and blank absorbances were read at 630 nm. The absorbance of the blank was subtracted from that of the sample and the α-amylase inhibitory activity was expressed as acarbose equivalents (mmol ACE/g extract).

For α-glucosidase inhibitory activity assay: Sample solution was mixed with glutathione (50 µL), α-glucosidase solution (from Saccharomyces cerevisiae, EC 3.2.1.20, Sigma) (50 µL) in phosphate buffer (pH 6.8) and PNPG (4-N-trophenyl-α-D-glucopyranoside, Sigma) (50 µL) in a 96-well microplate and incubated for 15 min at 37 °C. Similarly, a blank was prepared by adding sample solution to all reaction reagents without enzyme (α-glucosidase) solution. The reaction was then stopped with the addition of sodium carbonate (50 µL, 0.2 M). The sample and blank absorbances were read at 400 nm. The absorbance of the blank was subtracted from that of the sample and the α-glucosidase inhibitory activity was expressed as acarbose equivalents (mmol ACE/g extract).

6. Assay of wine extracts anti-inflammatory activity on LPS-stimulated macrophage

Cells were maintained in RPMI-1640 medium supplemented with 2 mM L-glutamine and 100 U/mL streptomycin-penicillin and 10% heat-inactivated fetal bovine serum (Sigma Aldrich, St. Louis, MO, USA) at 37°C with 5% CO_2. THP-1 cells were plated in 6-well culture plates at 1×106 cells/well and were differentiated to macrophages using 100 µg/mL phorbol-12-myristate-13-acetate (PMA, St. Louis, MO, USA) for 24 h with serum-free RPMI-1640 at 37°C. After 72 h the cells were treated with LPS at a final concentration of 0.1 µg/mL to stimulate cytokine production and with wine extracts at 25, 50 and 100 µg/mL.

After 24 h of incubation, the supernatant was removed and centrifuged to remove any cell residues. The IL-6, IL-1β and TNF-α release was quantified using an ELISA, according to the manufacturer's protocol (R&D Systems, Minneapolis, MN, USA).

In parallel, the effects of wine extracts on the viability of the LPS-stimulated macrophages was assessed using the 3-(4,5-dimethylthiazol-2-yl)-2,5-diphenyltetrazolium bromide (MTT) assay. After 24 h of exposure to wine extracts at 25, 50 and 100 µg/mL, 20 µL of MTT (1 mg/mL in PBS) was added to each well and incubated continuously for 4 h at normal culture conditions. The cells were then treated with 100 µL of DMSO. The absorbance was measured at 570 nm using a microplate reader (Thermo MK3, Winosky, VT, USA). Data were expressed as a percentage of the value obtained for the solvent control (0.1% DMSO), which was set to 100%.

To reduce any variation from differences in cell density, the ELISA results were normalized to the MTT values. The concentration of cytokines of the positive control (cells only treated with LPS) was defined as 100%. All results from the tested wine extracts were then calculated as a percentage of the positive control.

7. Lipooxygenase (5-LOX) inhibition assay

This assay measures the hydroperoxides generated from incubating a 5-LOX enzyme with its substrate, arachidonic acid (AA). Nordihydroguaiaretic acid was used as the positive control. A volume of 10 µL each of test extracts and vehicle were pre-incubated with 90 µL 5-LOX enzyme in a 96-well plate. The reaction was initiated by addition of 10 µL 1 mM AA and the plate was shaken for 5 minutes. Then, 100 µL of chromogen from the test kit was added to stop enzymatic reaction and for color development. The plate was placed on a shaker for another five minutes and absorbance at 490 nm was measured using microplate reader.

8. Statistical analysis

Statistical analysis was performed using GraphPad Prism version 5.01 for Windows (GraphPad Software, San Diego, CA). Means ± standard deviations (SDs) were determined for each experimental group. The antioxidant and enzyme inhibitory effects were analyzed using one-way analysis of variance (ANOVA) ($p < 0.05$). For pharmacological *in vitro* evaluation, data were analyzed by ANOVA followed by a Newman-Keuls comparison multiple test to assess the concentration–response relationship. Statistical significance was set at $p<0.05$. Pearson's correlation was carried out to assess the relationship between total bioactive compounds (phenolic and flavonoid) and antioxidant capacity. The correlation analysis was performed by Statistical version 8.0. For trans-resveratrol quantification, One-way analysis of variance (ANOVA) and Tukey's multiple range method were used, considering differences at $p < 0.05$ as statistically significant. The analysis was performed with R v 3.6.1 statistical software.

9. HPLC-photodiode array (PDA) instrument configuration

A stock solution was prepared for each sample with 1 mg in 1 mL of eluent, and HPLC analyses were performed on a Waters liquid chromatograph equipped with a PDA detector and a C18 reversed-phase column to separate the mixture. The column was thermostated at 30 ± 1

°C, and the elution was performed using a water–acetonitrile mobile phase (93:7, with 3% acetic acid). The complete separation of standards was achieved in 60 min. The ultraviolet/visible (UV/Vis) acquisition wavelength was set in the range of 200-500 nm.

1. Uysal, S.; Zengin, G.; Locatelli, M.; Bahadori, M. B.; Mocan, A.; Bellagamba, G.; De Luca, E.; Mollica, A.; Aktumsek, A., Cytotoxic and enzyme inhibitory potential of two Potentilla species (P. speciosa L. and P. reptans Willd.) and their chemical composition. *Frontiers in pharmacology* **2017,** 8, 290.
2. Grochowski, D. M.; Uysal, S.; Aktumsek, A.; Granica, S.; Zengin, G.; Ceylan, R.; Locatelli, M.; Tomczyk, M., In vitro enzyme inhibitory properties, antioxidant activities, and phytochemical profile of Potentilla thuringiaca. *Phytochemistry Letters* **2017,** 20, 365-372.

Table S1. Characterization of phytochemicals in extracts of wine by HPLC–ESI–MS^n.

Peak Number	Retention time (min)	$[M-H]^-$ *m/z*	ESI-MS^n product ions *m/z* (%) base peak	Dealcoholized	Aqueous extract	Organic phase 2	Organic phase 3	Grape skin decoction	Grape skin Soxhlet	Grape skin microwave	Assigned identification
1	1.7	439	MS^2 [439]: 421 (26), 341 (24), 179 (100), 161 (55) MS^3 [439→179]: 161 (100), 143 (17), 125 (32)	✓	✓			✓	✓	✓	Disaccharide (H_2SO_4 adduct)
2	1.9	191	MS^2 [191]: 173 (94), 111 (100)	✓			✓	✓	✓	✓	Isocitric acid
3	2.1	311	MS^2 [311]: 179 (57), 149 (100), 135 (16)				✓				Caftaric acid isomer
4	2.6	191	MS^2 [191]: 173 (48), 111 (100)	✓	✓		✓	✓			Citric acid
5	3.1	169	MS^2 [169]: 125 (100)	✓	✓	✓	✓	✓	✓		Gallic acid
6	3.7	616	MS^2 [616]: 484 (56), 440 (100), 272 (79) MS^3 [616→440]: 272 (100), 254 (27)	✓	✓						2-S-glutathionyl-caffeoyltartaric acid
7	3.9	315	MS^2 [315]: 153 (100), 123 (20)	✓	✓			✓	✓	✓	Hydroxytyrosol

											hexoside
8	5.1	219	MS^2 [219]: 173 (100), 157 (9), 111 (94)<br>MS^3 [219→173]: 111 (100)	✓	✓	✓	✓				Ethyl citrate
9	5.8	311	MS^2 [311]: 179 (43), 149 (100), 135 (4)	✓		✓	✓				Caftaric acid isomer
10	6.1	443	MS^2 [443]: 237 (34), 179 (100)	✓	✓			✓	✓	✓	Unknown
11	6.6	577	MS^2 [577]: 451 (43), 425 (100), 407 (45), 289 (32)	✓		✓		✓	✓	✓	Procyanidin dimer
12	7.0	175	MS^2 [175]: 157 (63), 129 (27), 115 (100), 113 (64)	✓	✓	✓	✓				Isopropylmalic acid
13	8.0	325	MS^2 [325]: 163 (100), 119 (17)	✓	✓		✓				Coumaric acid-*O*-hexoside
14	8.5	289	MS^2 [289]: 245 (100), 205 (35), 203 (36)	✓	✓	✓		✓	✓	✓	Catechin
15	8.7	295	MS^2 [295]: 163 (100)<br>MS^3 [295→163]: 119 (100)	✓		✓	✓				Coutaric acid
16	9.4	325	MS^2 [325]: 193 (100)<br>MS^3 [325→193]: 149 (100), 134 (98)			✓					Ferulic acid-*O*-pentoside isomer
17	9.7	325	MS^2 [325]: 265 (100), 235 (81)<br>MS^3 [325→235]: 163 (100)<br>MS^4 [325→235→163]: 119 (100)	✓	✓		✓		✓		Coumaric acid-*C*-hexoside
18	10.2	325	MS^2 [325]: 193 (100)<br>MS^3 [325→193]: 193 (100), 149 (58)<br>MS^4 [325→193→149]:134 (100).	✓		✓			✓		Ferulic acid-*O*-pentoside isomer
19	10.4	577	MS^2 [577]: 451 (20), 425 (100), 407 (73), 289 (27)	✓		✓		✓	✓	✓	Procyanidin dimer
20	11.0	179	MS^2 [179]: 135 (100	✓							Caffeic acid

21	11.2	325	MS^2 [325]: 265 (75), 235 (100)<br>MS^3 [325→235]: 163 (100)<br>MS^4 [325→235→163]:119 (100)	✓	✓	✓	✓	✓	✓		Coumaric acid-*C*-hexoside
22	11.7	289	MS^2 [289]: 245 (100), 205 (66), 203 (26)	✓		✓		✓	✓	✓	Epicatechin
23	12.6	493 (+)	MS^2 [493]: 331 (100)	✓	✓	✓		✓	✓	✓	Malvidin-*O*-glucoside
24	13.3	366	MS^2 [366]: 204 (68), 186 (100), 142 (26)	✓				✓			Unknown
25	13.9	186	MS^2 [186]: 169 (20), 125 (100)		✓						Gallic acid derivative
26	14.6	197	MS^2 [197]: 169 (100)<br>MS^3 [197→169]: 125 (100)	✓		✓	✓				Ethyl gallate
27	15.5	479	MS^2 [479]: 317 (100)<br>MS^3 [479→317]: 179 (100)	✓		✓	✓	✓	✓	✓	Myricetin-*O*-hexoside
28	16.4	493	MS^2 [493]: 317 (100)<br>MS^3 [493→317]: 179 (100), 151 (72)	✓		✓	✓				Myricetin-*O*-glucuronide
29	16.6	479	MS^2 [479]: 317 (100)<br>MS^3 [479→317]: 179 (100)	✓	✓	✓	✓	✓	✓	✓	Myricetin-*O*-hexoside
30	17.8	551	MS^2 [551]: 479 (57), 317 (100)<br>MS^3 [493→317]: 271 (100), 179 (50), 151 (28)	✓		✓	✓		✓		Myricetin derivative
31	18.8	389	MS^2 [389]: 227 (100)	✓		✓	✓				Trans-piceid
32	20.0	551	MS^2 [551]: 479 (57), 317 (100)<br>MS^3 [493→317]: 271 (100), 179 (50), 151 (28)		✓	✓	✓	✓	✓	✓	Myricetin derivative
33	20.4	477	MS^2 [477]: 301 (100)<br>MS^3 [477→301]: 179 (45), 151 (100)	✓	✓	✓	✓				Quercetin-*O*-glucuronide

34	20.9	449	MS^2 [449]: 303 (100), 285 (73), 151 (10)<br>MS^3 [449→303]: 285 (100), 177 (31), 125 (6)	✓		✓					Taxifolin-*O*-deoxyhexoside
35	21.1	551	MS^2 [551]: 479 (57), 317 (100)<br>MS^3 [493→317]: 271 (100), 179 (50), 151 (28)					✓	✓	✓	Myricetin derivative
36	21.5	353	MS^2 [353]: 163 (12), 145 (100), 117 (12)	✓		✓	✓				Unknown
37	23.4	447	MS^2 [447]: 301 (100)<br>MS^3 [447→301]: 271 (90), 255 (66), 179 (100), 151 (43)					✓	✓	✓	Quercetin-*O*-deoxyhexoside
38	24.1	389	MS^2 [389]: 227 (100)	✓	✓	✓	✓				Cis-Piceid
39	24.6	447	MS^2 [447]: 301 (100)<br>MS^3 [447→301]: 271 (90), 255 (66), 179 (100), 151 (43)	✓		✓		✓	✓	✓	Quercetin-*O*-deoxyhexoside
40	25.2	507	MS^2 [507]: 507 (100), 345 (27), 344 (26)<br>MS^3 [507→344]: 330 (43), 317 (25), 316 (54), 315 (45), 302 (45), 301 (100), 273 (37), 269 (17), 241 (24)<br>MS^4 [507→344→273]: 241 (100)	✓		✓	✓	✓	✓	✓	Syringetin-*O*-hexoside
41	26.2	623	MS^2 [623]: 315 (100), 314 (33)<br>MS^3 [623→315]: 300 (100), 299 (79)					✓	✓	✓	Isorhamnetin-*O*-rutinoside
42	26.4	317	MS^2 [317]: 179 (100)<br>MS^3 [317→179]: 151 (100)			✓					Myricetin
43	27.0	655	MS^2 [655]: 509 (17), 501 (30), 475 (49), 34(16), 329 (100), 314 (11)<br>MS^3 [655→329]: 329(17), 315(14), 314 (100), 149(9)				✓	✓	✓	✓	Laricitrin-3-*O*-rhamnose-7-*O*-trihydroxycinnamic acid

			MS^4 [655→329→314]: 315(33), 314(62), 299(100), 298(19), 163(12)								
44	28.3	431	MS^2 [431]: 285 (100) MS^3 [431→285]: 257 (31), 255 (100)	✓		✓					Kaempferol-*O*-deoxyhexoside
45	28.3	723	MS^2 [723]: 667 (100) MS^3 [723→667]: 667 (100)	✓	✓	✓	✓	✓	✓	✓	Unknown
46	28.7	637	MS^2 [637]: 329 (100) MS^3 [637→329]: 284 (61), 314 (100)			✓	✓	✓	✓	✓	Unknown
47	28.7	301	MS^2 [301]: 257 (100) MS^3 [301→257]: 185 (100)			✓					Ellagic acid

Figure S1. Relative peak areas (%) and heat map obtained by HPLC-ESI-MS^n analysis of wine extracts.

Peak	Compound	Dealcoholized	Aqueous extract	Organic phase 2	Organic phase 3	Grape skin decoction	Grape skin Soxhlet	Grape skin microwave
1	Disaccharide	9.81	19.13	0.00	0.00	2.97	0.92	2.43
2	Isocitric acid	3.03	0.00	0.00	3.52	0.03	0.03	0.37
3	Caftaric acid	0.00	0.00	0.00	4.60	0.00	0.00	0.00
4	Citric acid	2.59	18.79	0.00	1.19	0.01	0.00	0.00
5	Gallic acid	8.73	2.13	6.78	5.03	0.06	0.04	0.00
6	Glutathionyl-caffeoyltartaric acid	3.19	5.84	0.00	0.00	0.00	0.00	0.00
7	Hydroxytyrosol hexoside	4.49	10.11	0.00	0.00	0.95	0.98	0.86
8	Ethyl citrate	1.96	1.33	1.93	6.77	0.00	0.00	0.00
9	Caftaric acid	4.53	0.00	6.91	4.10	0.00	0.00	0.00
10	Unknown	2.28	9.21	0.00	0.00	0.37	0.56	0.41
11	Procyanidin dimer	0.45	0.00	0.43	0.00	0.00	0.00	0.00
12	Isopropylmalic acid	5.21	1.69	6.07	15.63	0.00	0.00	0.00

13	p-Coumaric acid-*O*-hexoside	0.36	1.18	0.00	0.88	0.00	0.00	0.00
14	Catechin	2.91	0.97	3.06	0.00	2.03	2.46	0.82
15	Coutaric acid	2.01	0.00	5.16	0.50	0.00	2.56	0.00
16	Ferulic acid-*O*-pentoside	0.00	0.00	2.17	0.00	0.00	0.00	0.00
17	Coumaric acid-C-hexoside	2.65	4.62	0.00	4.72	0.00	0.00	0.00
18	Ferulic acid-*O*-pentoside	2.65	0.00	2.17	0.00	0.00	1.01	1.51
19	Procyanidin dimer	0.82	0.00	0.59	0.00	0.48	3.55	0.45
20	Caffeic acid	1.24	0.00	0.00	0.00	0.00	0.00	0.00
21	Coumaric acid-*C*-hexoside	2.15	6.42	0.50	6.60	0.19	1.15	0.00
22	Epicatechin	1.17	0.00	0.02	0.00	2.32	2.44	2.21
23	Malvidin-*O*-glucoside	0.68	4.26	2.94	0.00	49.17	32.52	49.88
24	Unknown	2.43	0.00	0.00	0.00	0.64	0.00	0.00
25	Gallic acid derivative	0.00	2.08	0.00	0.00	0.00	0.00	0.00
26	Ethyl gallate	10.04	0.00	18.05	1.17	0.00	0.00	0.00
27	Myricetin-*O*-hexoside	2.70	0.00	3.02	5.61	1.39	5.26	0.84
28	Myricetin-*O*-glucuronide	0.49	0.00	0.44	1.68	0.00	0.00	0.00
29	Myricetin-*O*-hexoside	3.01	1.27	3.24	5.98	4.79	6.06	5.56
30	Myricetin derivative	0.20	0.00	0.64	4.89	0.00	0.20	0.00
31	Trans-Piceid	1.55	0.00	0.99	3.02	0.00	0.00	0.00
32	Myricetin derivative	0.00	0.91	0.75	5.01	6.51	1.74	2.43
33	Quercetin-*O*-glucuronide	4.05	0.45	8.94	6.52	0.00	0.00	0.00
34	Taxifolin-*O*-deoxyhexoside	0.84	0.00	1.44	0.00	0.00	0.00	0.00
35	Myricetin derivative	0.00	0.00	0.00	0.00	0.00	5.74	0.00
36	Unknown	1.53	0.00	2.48	1.38	0.00	0.00	0.00
37	Quercetin-*O*-deoxyhexoside	0.00	0.00	0.00	0.00	0.97	0.81	1.12
38	Cis-Piceid	3.02	0.67	6.12	2.95	0.00	0.00	0.00
39	Quercetin-*O*-deoxyhexoside	2.09	0.00	2.80	0.00	5.76	7.18	0.83
40	Syringetin-*O*-hexoside	2.04	0.00	2.81	2.84	0.00	1.72	1.34
41	Isorhamnetin-*O*-rutinoside	0.00	0.00	0.00	0.00	2.52	2.04	3.13

42	Myricetin	0.00	0.00	6.01	0.00	0.00	0.00	0.00
43	Laricitrin derivative	0.00	0.00	0.00	1.16	1.01	4.37	8.80
44	Kaempferol-*O*-deoxyhexoside	0.33	0.00	0.48	0.00	0.00	0.00	0.00
45	Unknown	2.78	8.95	0.35	2.72	4.62	4.12	3.47
46	Unknown	0.00	0.00	0.67	1.53	13.21	12.52	13.56
47	Ellagic acid	0.00	0.00	2.05	0.00	0.00	0.00	0.00

Figure S2. Calibration curve obtained by linear regression of areas against the resveratrol concentration (µg).

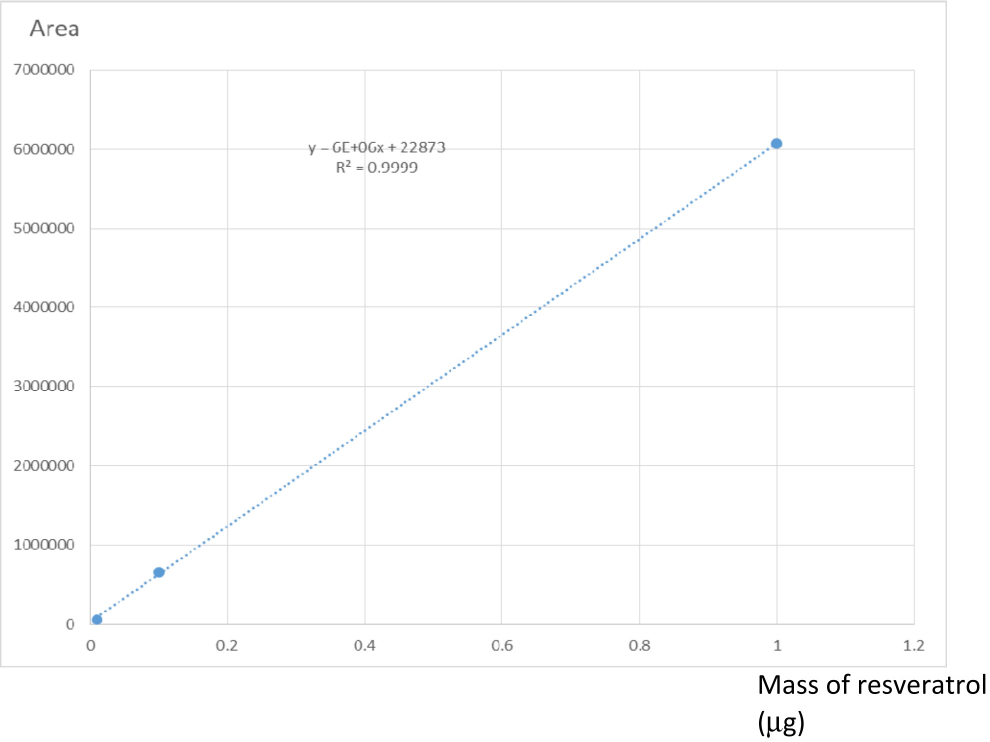

x (µg)	Areas
1	6067770
0.1	655537
0.01	57966
